# Modulation of NF-κB and TLR Signaling Pathways and Complement Components in Ovine Maternal Thyroid During Early Pregnancy

**DOI:** 10.3390/ijms27114791

**Published:** 2026-05-26

**Authors:** Yaqi Zhang, Jingjing Li, Fei Yang, Chenxu Wu, Leying Zhang, Ling Yang

**Affiliations:** School of Life Sciences and Food Engineering, Hebei University of Engineering, Handan 056038, China; 18632804307@163.com (Y.Z.); 15137164507@163.com (J.L.); 18232076022@163.com (F.Y.); wuchenxu112@163.com (C.W.); zhangly056000@126.com (L.Z.)

**Keywords:** ewe, IκB family, immune regulation, protein and mRNA expression, thyroid function

## Abstract

Pregnancy modulates the function of the thyroid gland to facilitate maternal immune tolerance, and nuclear factor kappa B (NF-κB) subunits, the IκB family, and toll-like receptors (TLRs) and complement signaling pathways may be implicated in maternal thyroid immunoregulation. However, it is unclear whether early pregnancy modulates the expression of NF-κB subunits, the IκB family, TLRs, and complement components in the maternal thyroid. The objective of this study was to analyze the effects of early pregnancy on the expression of genes and proteins of these signaling pathways in the maternal thyroid in ewes. In this study, ovine thyroids (*n* = 6 for each group) were sampled on day 16 of the estrous cycle (N16) and on days 13, 16, and 25 of pregnancy (P13, P16, and P25) with one conceptus. The ewes had an average weight of 41 kg and a body condition score of 3. The mRNA and protein expression of the NF-κB subunits and IκB family were analyzed by RT-qPCR, western blot, and immunohistochemistry. The results showed that the expression of all NF-κB subunits, the IκB family, and TLRs, as well as C1q, C1r, C2, C4a, C5b, and C9, peaked at P16 among these four stages (*p* < 0.05). In addition, C1s expression was greater at N16 and P16 than at P13 and P25 (*p* < 0.05), and C3 expression was stronger at P16 and P25 compared to N16 and P13 (*p* < 0.05). In conclusion, early pregnancy modulates the expression of NF-κB subunits, the IκB family, TLRs, and complement components in the ovine thyroid at both the mRNA and protein levels, which may be essential for maternal thyroid adaptation to pregnancy and beneficial for the prevention of pregnancy-related thyroid diseases in ewes.

## 1. Introduction

During pregnancy in humans, human chorionic gonadotropin regulates the secretion of maternal thyroid hormones by thyrotropin through the hypothalamic–pituitary–thyroid axis to meet metabolic changes [1]. The size of the maternal thyroid gland increases to compensate for the increasing demand for thyroid hormones during pregnancy, which is essential for optimal pregnancy outcomes [2]. In addition, the thyroid gland produces thyroid hormones thyroxine and triiodothyronine, which have effects on immune cells to modulate the innate and adaptive immune responses [3]. Pregnancy-related hormonal changes modulate the activity of innate immune cells, which have a significant influence on the function of the thyroid gland. Thyroid disorders, for example, autoimmune thyroid disease (AITD), can affect fertility and cause adverse outcomes during pregnancy, so maintaining thyroid immune equilibrium during pregnancy is essential [4].

The nuclear factor kappa B (NF-κB) family comprises five members, including NF-κB1, NF-κB2, RelA, c-Rel, and RelB, and the expression of the NF-κB family in fetal and maternal tissues is modulated at the outset and the end of pregnancy [5]. RelA expression is downregulated in human anaplastic thyroid carcinoma cell lines after sesquiterpene lactone cynaropicrin treatment, suggesting that RelA has a proliferative effect on thyroid cells [6]. In addition, NF-κB2 participates in cell invasion and cell migration of thyroid cells in thyroid cancer carcinogenesis [7]. The activities of the NF-κB transcription factor are regulated by ikappaB (IκB) and IκB kinase (IKK), which include B-cell lymphoma-3 (BCL-3), IκBα, IκBβ, IκBε, IκBζ, and IκBδ, as well as NEMO/IKKγ [8]. Furthermore, activation of NF-κB in human thyroid neoplasms is resistant to drug-induced apoptosis, but stable expression of IκBα enhances sensitivity to drug-induced apoptosis in mice [9]. IκBα polymorphisms have a close relation with susceptibility to Graves’ disease, an autoimmune disease of the thyroid, suggesting that IκBα is involved in the development of thyroid autoimmunity in humans [10].

Toll-like receptors (TLRs) modulate the activation of common and specific signaling pathways to shape immune responses, which play a dual role in immune defense and disease pathogenesis [11]. TLR2 plays a protective role against Schistosoma japonicum peptide in excessive iodine-induced pyroptosis in thyroid follicular epithelial cells by inhibiting reactive oxygen species/mitogen-activated protein kinases/NF-κB signaling [12]. In addition, the TLR signaling pathway member, myeloid differentiation primary response gene 88 (MyD88), is implicated in bacterial infection-induced complex dysfunctions of the hypothalamic–pituitary–thyroid axis, suggesting that MyD88 participates in the modulation of thyroid gland function in mice [13]. Additionally, complement components mainly include complement component 1 (C1) complex (C1qC1r2s2), C3, C2, C4, C5, and C9. They are expressed in immune and non-immune cells and tissues to control normal cell turnover and responses to infectious and non-infectious stimuli [14]. Moreover, complement components C3a, C4d, and soluble C5b-9 are expressed in thyroid tissues from patients with Hashimoto’s thyroiditis and papillary thyroid cancer, which are involved in the pathogenesis of these thyroid diseases in humans [15]. Therefore, the TLR signaling pathway and complement system are implicated in thyroid immunoregulation in humans. Compared with mice, sheep usually have more singleton pregnancies, a longer gestation period, and organs with sizes similar to those of humans. Thus, sheep may be used as an animal model for humans.

Pregnancy recognition signals (interferon-tau (IFNT) in ruminants and chorionic gonadotropin in humans) and progesterone reprogram the maternal immune cell function in the maternal uterus and peripheral tissues to help conceptuses evade maternal immunological detection during pregnancy [16]. In addition, during early pregnancy in sheep, the expression of interferon-stimulated genes in the maternal thyroid and corpus luteum is modulated by IFNT [17,18]. However, the simultaneous regulation of the NF-κB, TLR, and complement pathways in the maternal thyroid during early pregnancy remains unexplored. Therefore, we hypothesize that early pregnancy may have effects on the NF-κB and TLR signaling pathways and complement components in the maternal thyroid. This study aimed to detect the expression of genes and proteins of NF-κB signaling (NF-κB1, NF-κB2, RelA, RelB, and c-Rel), the IκB family (BCL-3, IκBα (NFKBIA), IκBβ (NFKBIB), IκBε (NFKBIE), IKKγ (IKBKG), IκBδ (NFKBID) and IκBζ (NFKBIZ)), TLR signaling (TLR2, TLR3, TLR4, TLR5, MyD88, tumor necrosis factor receptor-associated factor 6 (TRAF6), and interleukin 1 receptor-associated kinase 1 (IRAK1)), and complement components (C1q, C1r, C1s, C2, C3, C4a, C5b, and C9) in the maternal thyroid during early pregnancy in ewes. The results of this study will be helpful for understanding maternal thyroid adaptation to pregnancy and preventing pregnancy-related thyroid diseases in ruminants, which may provide a reference for human-related research.

## 2. Results

### 2.1. The Expression of the NF-κB Signaling Pathway in the Maternal Thyroid

**Figure 1:**
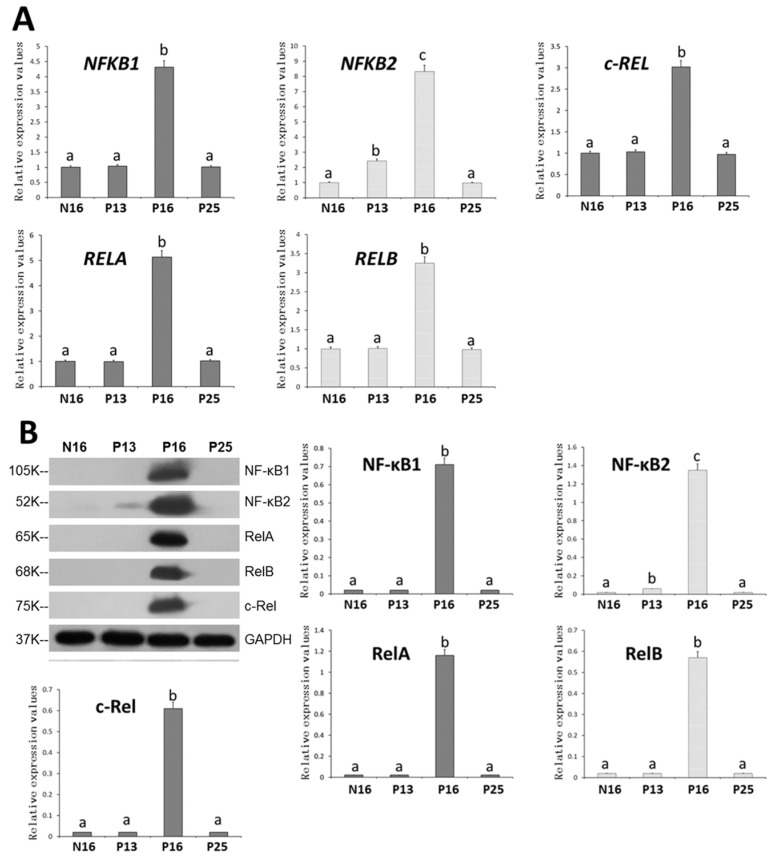

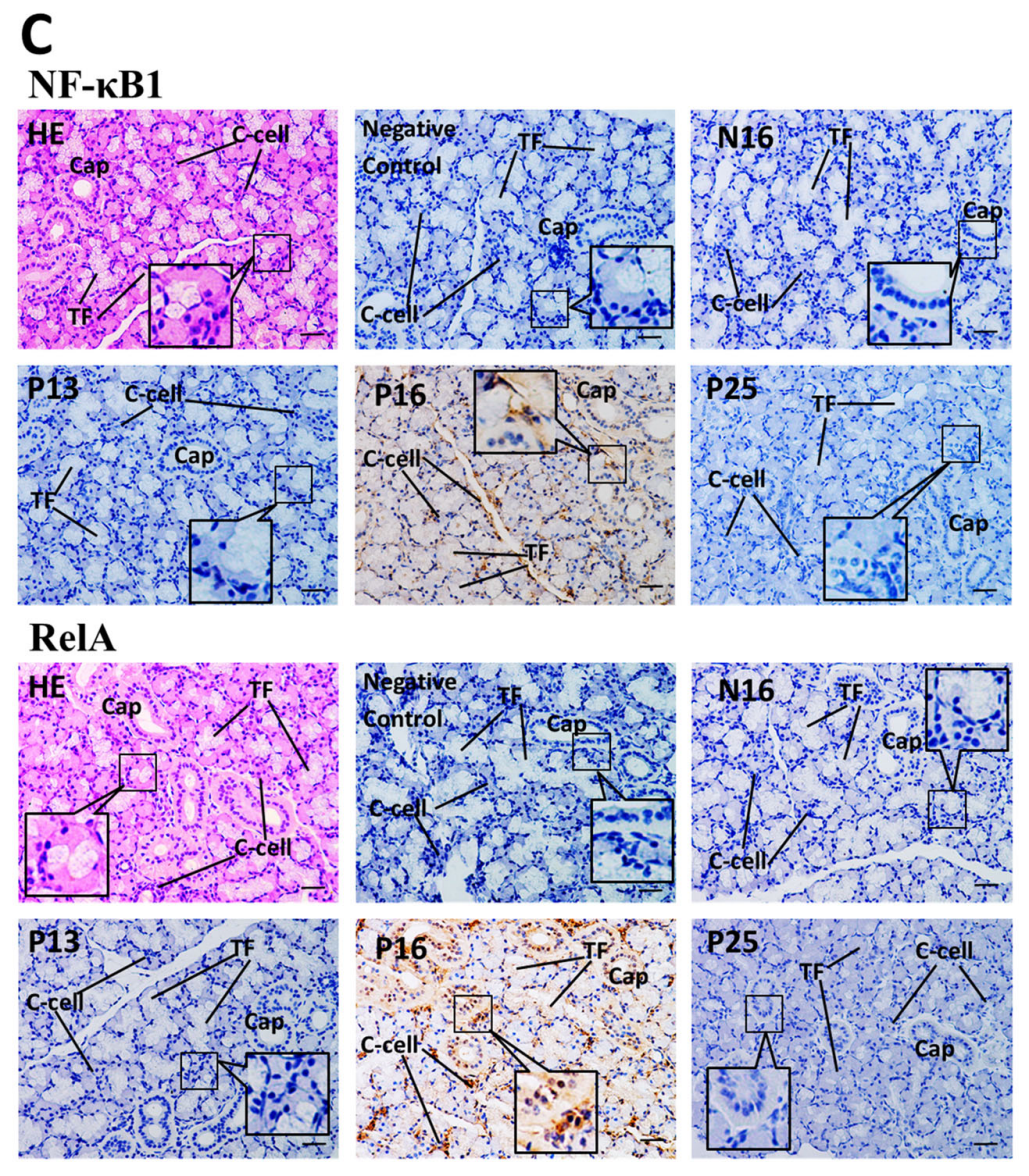
Expression of NF-κB subunits in the thyroid. (**A**). NF-κB subunit mRNA expression values. (**B**). NF-κB subunit protein expression values. Significant differences (*p <* 0.05) are indicated by different letters. (**C**). Representative immunohistochemical localization of the NF-κB1 and RelA proteins in the thyroid. The thyroid follicle (TF), containing colloid in its lumen, is lined predominantly by parafollicular cells (C-cells). Cap = capillary; N16 = day 16 of the estrous cycle; P13 = day 13 of pregnancy; P16 = day 16 of pregnancy; P25 = day 25 of pregnancy. Bar = 50 µm.

Figure 1A,B showed that early pregnancy had similar effects on the mRNA and protein expression of all five members of the NF-κB signaling pathway. The expression values of all five members peaked at P16 (*p* < 0.05), and there was no significant difference in the expression of NF-κB1 (*NF-κB1*), RelA (*RELA*), RelB (*RELB*), and c-Rel (*REL*) among N16, P13, and P25 (*p* > 0.05). In addition, the values of NF-κB1 (*NF-κB1*) gene and protein were greater at P13 than at N16 and P25 (*p* < 0.05). In addition, NF-κB1, RelA, RelB, and c-Rel proteins were almost undetectable at N16, P13, and P25. NF-κB2 protein was almost undetectable at N16 and P25.

Immunohistochemical results (Figure 1C) for representative proteins of the NF-κB signaling pathway showed that NF-κB1 and RelA proteins were strongly located in parafollicular cells, also known as thyroid medullary cells (C-cells), and capillaries. The staining intensities of NF-κB1 and RelA proteins were the highest in thyroids from P16. In addition, there was almost no staining in other stages.

### 2.2. The Expression of the IκB Family in the Maternal Thyroid

**Figure 2:**
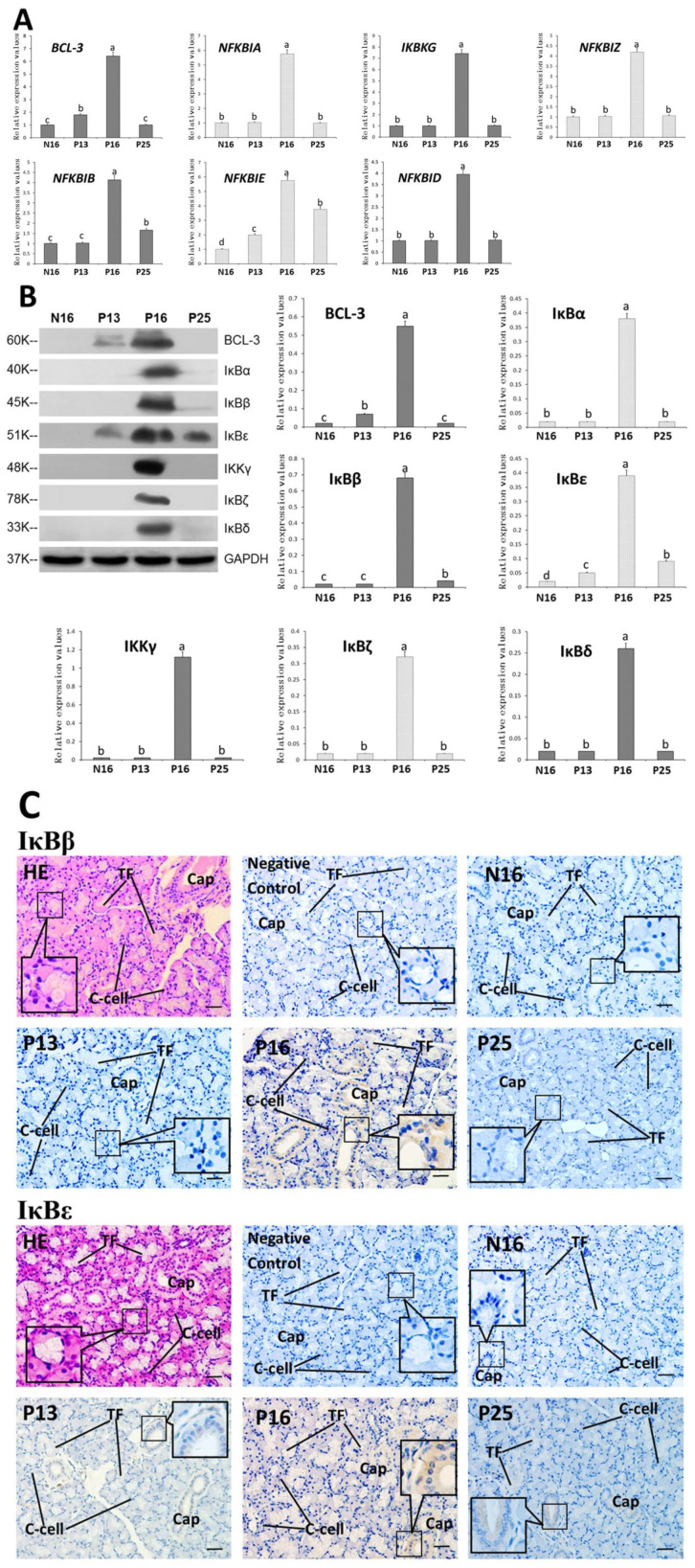
IκB family in the thyroid. (**A**). IκB family mRNA expression values. (**B**). IκB family protein expression values. Significant differences (*p <* 0.05) are indicated by different letters. (**C**). Representative immunohistochemical localization of IκBβ and IκBε proteins in the thyroid. The thyroid follicle (TF), containing colloid in its lumen, is lined predominantly by parafollicular cell (C-cells). Cap = capillary; N16 = day 16 of the estrous cycle; P13 = day 13 of pregnancy; P16 = day 16 of pregnancy; P25 = day 25 of pregnancy. Bar = 50 µm.

The expression patterns of IκB family mRNA and proteins were similar (Figure 2A,B). The expression values of the IκB family peaked at P16, and there was no significant difference in the expression values of IκBα (*NFKBIA*), IKKγ (*IKBKG*), IκBδ (*NFKBID*), and (IκBζ) *NFKBIZ* among N16, P13, and P25 (*p* > 0.05). In addition, the values of BCL-3 (*BCL-3*) were lower at N16 and P25 than at P13 (*p* < 0.05), and those of IκBβ (*NFKBIB*) and IκBε (*NFKBIE*) were greater at P25 compared to N16 and P13 (*p* < 0.05). Furthermore, BCL-3 protein was almost undetectable at N16 and P25, as were IκBα, IκBβ, IKKγ, IκBζ, and IκBδ proteins at N16, P13, and P25, as well as IκBε protein at N16.

Immunohistochemical results (Figure 2C) for representative proteins of the IκB family revealed that IκBβ and IκBε proteins were strongly localized in C-cells and capillaries, and the staining intensities of NF-κB1 and RelA proteins were the highest in thyroids from P16. In addition, there was almost no staining for IκBβ protein in other stages. Furthermore, the staining intensities of IκBε protein were weak at P13 and P25, and there was almost no staining at N16.

### 2.3. The Expression of the TLR Signaling Pathway in the Maternal Thyroid

**Figure 3:**
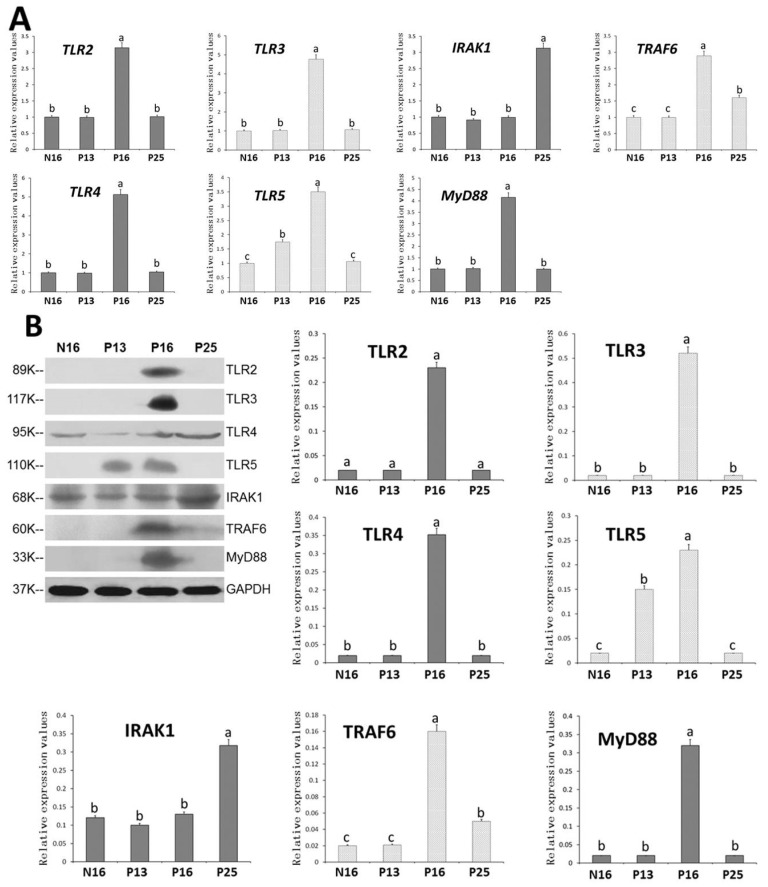

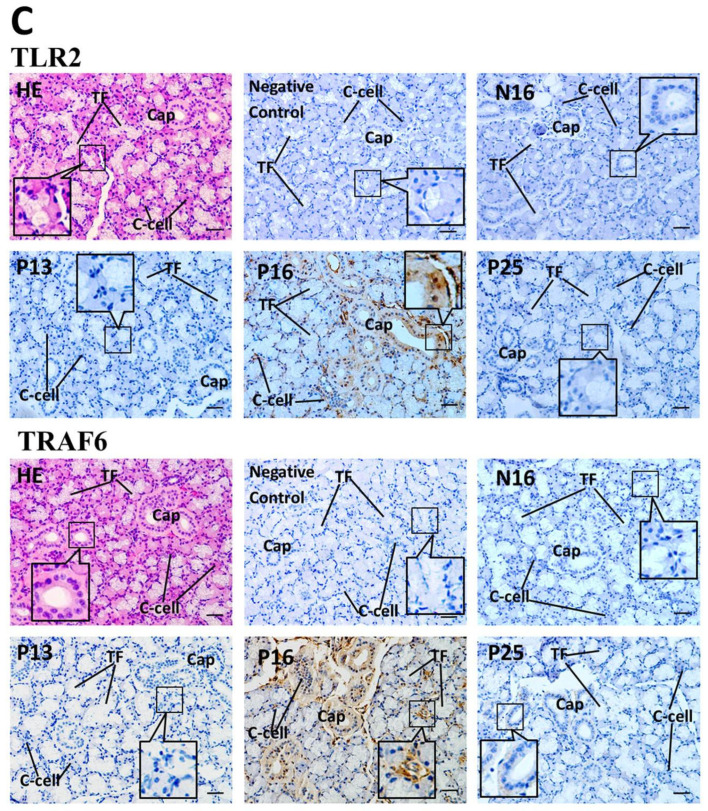
Expression of the TLR signaling pathway in the thyroid. (**A**). Numbers of TLR signaling pathway in mRNA expression. (**B**) Numbers of TLR signaling pathway in protein expression. Significant differences (*p <* 0.05) are indicated by different letters. (**C**). Representative immunohistochemical localization of TLR2 and TRAF6 proteins in the thyroid. The thyroid follicle (TF), containing colloid in its lumen, is lined predominantly by parafollicular cells (C-cells). Cap = capillary; N16 = day 16 of the estrous cycle; P13 = day 13 of pregnancy; P16 = day 16 of pregnancy; P25 = day 25 of pregnancy. Bar = 50 µm.

The expression patterns of the TLR signaling pathway in protein and mRNA (Figure 3A,B) were almost similar. The expression values of TLR2, TLR3, TLR4, TLR5, MyD88, and TRAF6 peaked at P16 (*p* < 0.05). There was no significant difference in the expression levels of TLR2, TLR3, TLR4, and MyD88 among N16, P13, and P25 (*p* > 0.05). In addition, the expression value of TLR5 was greater at P13 compared to N16 and P25 (*p* < 0.05), and the expression level of TRAF6 was greater at P25 compared to N16 and P13 (*p* < 0.05). However, *IRAK1* mRNA and protein levels peaked at P25 (*p* < 0.05), and there was no significant difference among N16, P13, and P16 (*p* > 0.05). Additionally, TLR2, TLR3, TLR4, MyD88, and TRAF6 proteins were almost undetectable at N16, P13, and P25, as well as TLR5 protein at N16 and P25.

Immunohistochemical results (Figure 3C) for representative proteins of the TLR signaling pathway indicated that TLR2 and TRAF6 proteins were strongly localized in C-cells and capillaries, and the staining intensities of TLR2 and TRAF6 proteins were the highest at P16. In addition, there was almost no staining in other stages.

### 2.4. Modulation of Complement Components in the Maternal Thyroid During Early Pregnancy

**Figure 4:**
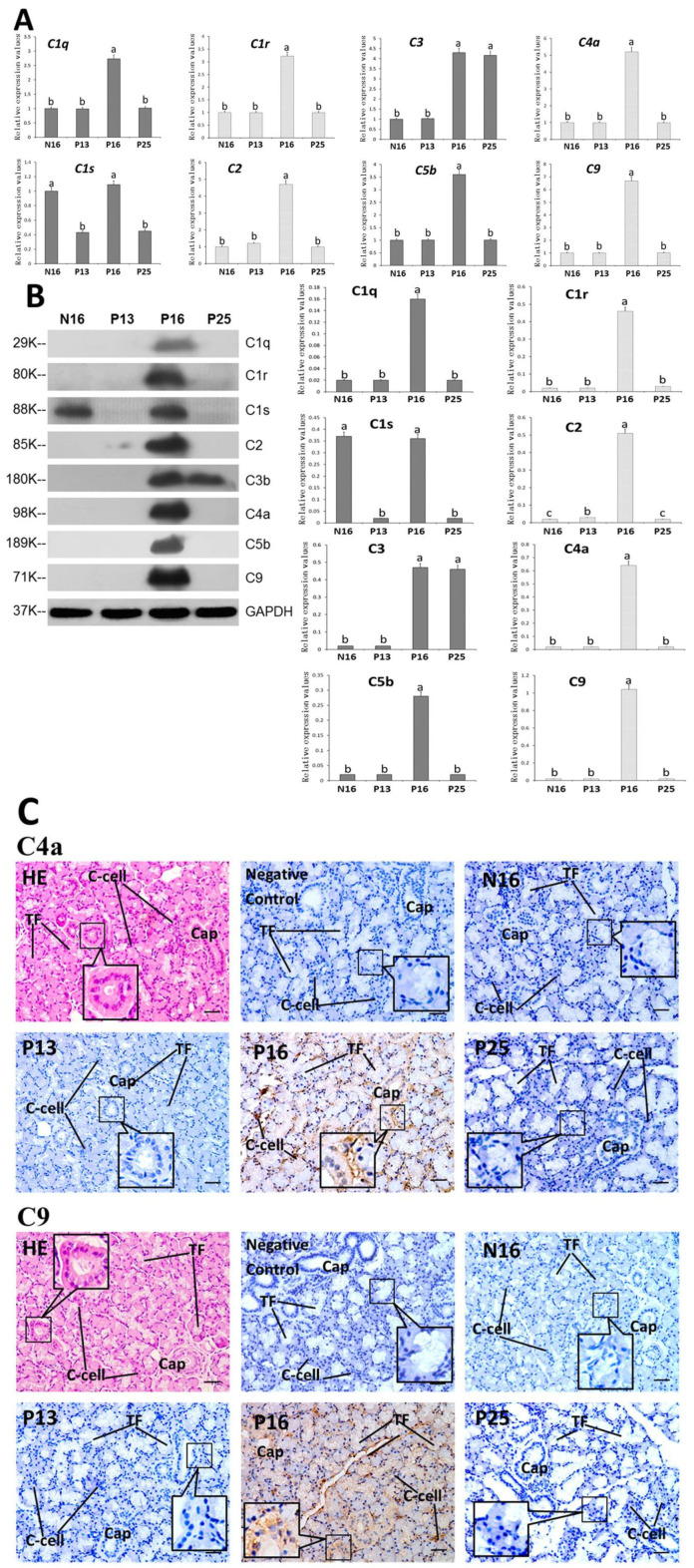
Complement components in the thyroid. (**A**). Complement component mRNA expression values. (**B**). Complement component protein expression values. Significant differences (*p <* 0.05) are indicated by different letters. (**C**). Representative immunohistochemical localization of C4a and C9 proteins in the thyroid. The thyroid follicle (TF), containing colloid in its lumen, is lined predominantly by parafollicular cells (C-cells). Cap = capillary; N16 = day 16 of the estrous cycle; P13 = day 13 of pregnancy; P16 = day 16 of pregnancy; P25 = day 25 of pregnancy. Bar = 50 µm.

The expression patterns of complement components in both protein and mRNA (Figure 4A,B) were almost similar (Figure 1A,B). The expression levels of C1q, C1r, C2, C4a, C5b and C9 peaked at P16 (*p* < 0.05). C1q, C1r, C2, C4a, C5b and C9 proteins were almost undetectable at N16, P13, and P25. In addition, the expression value of C1s was greater at N16 and P16 compared to P13 and P25 (*p* < 0.05), and the expression level of C3 was greater at P16 and P25 compared to N16 and P13 (*p* < 0.05). Furthermore, C1s protein was almost undetectable at P13 and P25, and C3 protein was almost undetectable at N16 and P13.

Immunohistochemical results (Figure 4C) for representative proteins of complement components showed that C4a and C9 proteins were strongly localized in C-cells and capillaries, and the staining intensities of C4a and C9 proteins were the highest at P16. In addition, there was almost no staining in other stages.

## 3. Discussion

### 3.1. Pregnancy Modulates the NF-κB Signaling Pathway in the Maternal Thyroid

In this study, early pregnancy induced peaks at both the mRNA and protein levels of NF-κB1 (*NFKB1*) and RelA (*RELA*) in the ovine thyroid at P16, and NF-κB1 and RelA proteins were strongly localized in the thyroid C-cells and capillaries. NF-κB1 plays an essential role in interferon (IFN)-α treatment for multiple myeloma patients [19]. In addition, NF-κB1 expression is upregulated in the maternal thymus during early pregnancy [20], and IFNT reaches the maximum abundance at P16 in sheep [21]. Additionally, RelA modulates transcriptional activity and increases the binding of NF-κB1 to DNA, which is implicated in the development of thyroid carcinoma through a canonical pathway in humans [22]. There is an increase in RelA expression in the uterine epithelium, which regulates immune tolerance during early pregnancy in bovines [23]. Moreover, RelA is involved in the transcription of thyroid peroxidase to regulate thyroid autoimmunity [9].

Our data indicated that there were peaks at both the mRNA and protein levels of NF-κB2 (*NFKB2*) and RelB (*RELB*) in the thyroid. NF-κB2 and RelB are associated with the corticotropin-releasing hormone produced in the full-term human placenta [24]. In addition, NF-κB2 is involved in long non-coding RNA function in thyroid cancer carcinogenesis, including DNA synthesis, cell invasion, and cell migration of thyroid cells [7]. However, the *NFKB2* variant has an adverse impact on the production of IFN-ω with common variable immune deficiency in humans [25]. Furthermore, the expression of RelB in thyroid cancer cell lines and papillary thyroid carcinoma indicates that the alternative NF-κB pathway is involved in tumor progression of thyroid cancers [26].

Our results showed that c-Rel protein and mRNA expression peaked in the maternal thyroid at P16. The expression of c-Rel in the myometrium and myometrial biopsies is decreased, which contributes to myometrial quiescence and pregnancy maintenance in humans [27]. In addition, as an essential member of the NF-κB family, c-Rel is significantly weaker in thyroid cancer cells treated with emodin, suggesting that c-Rel participates in cell proliferation, apoptosis, and carcinogenicity in thyroid cells [28]. Additionally, the expression of IFN regulatory factor 4, a member of the IFN family, induced by mitogen is c-Rel dependent in lymphocytes [29].

In short, the peak of all five members at P16 may be associated with the peak of IFNT, but the downregulation at P25 may benefit immune regulation of the maternal thyroid gland.

### 3.2. Early Pregnancy Affects the Expression of the IκB Family in the Maternal Thyroid

This study revealed that mRNA and protein expression of BCL3 and IκBε (*NFKBIE*) peaked at P16, and IκBε protein was strongly localized in the thyroid C-cells and capillaries. BCL3 is initially identified as a proto-oncogene and also regulates NF-κB-dependent gene transcription through DNA binding to NF-κB1 or NF-κB2 [30]. In addition, the mRNA and protein levels of BCL3 were increased, stimulated by thyroid hormone T3, suggesting that BCL3 plays a key role in T3-induced hepatocyte proliferation in rats [31]. Furthermore, the *BCL3* gene is downregulated in patients with low-risk thyroid cancer after treatment with microwave ablation [32]. Moreover, the IκBε expression peaks at P16 in the maternal liver, suggesting that the peak of IκBε expression is involved in NF-κB suppression and related to the IFNT secretion in ewes [33]. *NFKBIE* is involved in macrophage interactions with CD8^+^ T cells and activating relevant immune pathways to influence the development of Graves’ disease in humans [34].

It was shown in this study that early pregnancy induced peaks at both the mRNA and protein levels of IκBα (*NFKBIA*) and IκBβ (*NFKBIB*) at P16 in the thyroid, and IκBβ protein was strongly localized in the thyroid C-cells and capillaries. Degradation of IκBα contributes to the activation of the NF-κB signaling pathway, which leads to proliferation and migration in patients with papillary thyroid cancer [35]. In addition, upregulation of IFN-λ is positively correlated with the expression of IκBα in cells with low-level infection by SARS-CoV-2 [36]. Additionally, there is a peak in IκBα expression at P16 in the maternal lymph nodes, which plays a key role in the establishment of maternal immune tolerance in ewes [37]. Moreover, IκBβ expression in mRNA and protein levels peaks at P16 in the maternal liver, which is associated with the IFNT from the conceptus and pregnancy recognition [33]. On the other hand, treatment with polyriboinosinic polyribocytidylic acid stimulates mRNA expression of both *IFN-α* and *IκBβ* in the brain in immunologically induced fatigue in rats [38].

Protein and mRNA expression of thyroid IκBδ (*NFKBID*) and IκBζ (*NFKBIZ*) peaked at P16 in this study. There is an upregulation of IκBδ in the uterine tissue during implantation/placentation, which contributes to restricting embryo arrestment in mice [5]. In addition, during early gestation, IκBδ expression is upregulated at P16 in the maternal spleen, which is involved in the modulation of maternal immune functions in ewes [37]. IκBδ protein is expressed in T cells and plays a vital role in IFN-γ production, and IκBζ is the most similar homolog of IκBδ [39]. Furthermore, the expression of IκBζ protein and mRNA in lymph nodes peaks at P16, which is associated with the immune regulation of the maternal lymph nodes in ewes [37]. Moreover, dimethyl itaconate and 4-octyl itaconate can inhibit IκBζ and IFN-β secretion, but lipopolysaccharide stimulates IκBζ and IFN-β secretion in macrophages [40].

The data from this study showed that mRNA and protein expression of IKKγ (*IKBKG*) peaked at P16. IKKγ plays a critical role in the activation of NF-κB and is also necessary for thyroid differentiation in mice [41]. However, in humans, the upregulation of *IKBKG* gene expression in maternal and fetal blood is related to preeclampsia development [42]. In addition, a previous study reported that IKKγ expression in the maternal liver peaked at P16, suggesting that IKKγ is involved in the regulation of the hepatic inflammatory response and pregnancy establishment in ewes [33].

In summary, the peaks of the IκB family may be associated with the peak of IFNT at P16 and implicated in the modulation of the thyroid hormone secretion and pregnancy recognition.

### 3.3. Changes in the Expression of the TLR Signaling Pathway in the Maternal Thyroid

This study showed that early pregnancy induced peaks at both the mRNA and protein levels of TLR2 and TLR3 in the ovine thyroid at P16, and TLR2 protein was strongly localized in the thyroid C-cells and capillaries. In addition, early pregnancy (day 18 of pregnancy) upregulates *TLR2* expression in both trophoblasts and peripheral cells, which is implicated in immunological regulation at the maternal-fetal interface in ewes [43]. However, activation of the TLR2/phosphoinositide 3-kinase pathway promotes the proliferation of thyroid carcinoma cells and leads to local immune inflammatory response [44]. Additionally, there is greater expression of TLR3 in the thyrocytes of patients with Hashimoto’s thyroiditis and Graves’ disease [45]. TLR3 is usually expressed in maternal and fetal cells, but its expression is greater in preeclamptic pregnancies [46]. Moreover, the expression of *TLR3* mRNA and protein peaks at P16, which is associated with the modulation of maternal thymic immunity in sheep [47].

Our data showed that there were peaks at both the mRNA and protein levels of TLR4 and TLR5 in the thyroid. The TLR4/NF-κB signaling pathway is associated with thyroid tissues in rats with autoimmune thyroiditis [48]. In addition, the mRNA and protein levels of TLR4 peak in the maternal lymph node at P16, playing a key role in modulating the innate immune response of maternal lymph nodes in sheep [49]. Furthermore, TLR4 signaling can drive IFN-β production at endosomes originating from the intracellular pool of immune cells [50]. Moreover, there is moderate expression of TLR5 protein in thyroid nodular hyperplasia tissue and significant downregulation of TLR5 protein in papillary thyroid carcinoma and anaplastic thyroid carcinoma tissues in humans [51]. On the other hand, the expression of *TLR5* mRNA peaks in the porcine endometrium on day 15 of pregnancy, which is associated with increasing doses of progesterone and maternal immune modulation during placental development in pigs [52]. 

Our results revealed that protein and mRNA expression of TRAF6 and MyD88 peaked in the maternal thyroid at P16, and TRAF6 protein was strongly localized in the thyroid C-cells and capillaries. Macrophage M1 polarization drives recurrent spontaneous abortion, which can be prevented by TRAF6 [53]. In addition, TRAF6 is involved in NF-κB-mediated IFN-β production during virus infection in DF-1 and HEK293T cells [54]. TRAF6-mediated pathways are also implicated in the suppression of thyroid cancer angiogenesis and metastasis induced by emodin [55]. Moreover, the level of MyD88 protein in cord blood samples is higher in pregnant women compared to nonpregnant females, suggesting that MyD88 protein plays a key role in fighting against various pathogen infections [56]. MyD88 expression in mRNA and protein levels peaks at P16 in the maternal duodenum, which is associated with early pregnancy signals and intestinal homeostasis [57]. On the other hand, MyD88 signaling mediates thyroid gland function in mice with nonthyroidal illness [13].

This study revealed that mRNA and protein expression of IRAK1 peaked at P25. Primary human myometrial cells transfected with IRAK1 showed a decrease in the expression of cytokines, chemokines and adhesion molecules, suggesting that IRAK1 is involved in the regulation of pro-inflammatory and pro-labor mediators in human primary myometrial cells [58]. In addition, IRAK1 polymorphisms are related to the risks of AITD in humans [59].

In short, the peaks of TLR2, TLR3, TLR4, TLR5, and MyD88 at P16 may be related to the peak of IFNT and pregnancy recognition. However, the upregulation of IRAK1 at P25 may contribute to the modulation of thyroid hormone secretion and pregnancy maintenance.

### 3.4. Complement Components in the Maternal Thyroid During Early Pregnancy

This study revealed that early pregnancy induced peaks at both the mRNA and protein levels of C1q and C1R at P16 in the thyroid, while C1s expression was greater at N16 and P16 compared to P13 and P25. The upregulation of complement C1q protein at day 18 after insemination is related to the early pregnancy signal (IFNT), which can be used as an ideal marker for early pregnancy diagnosis in cows [60]. In addition, *C1q* mRNA and protein expression peaks at P16 in the maternal thymus and lymph nodes, which is associated with maternal immune regulation and pregnancy recognition in ewes [61,62]. Anti-C1q antibody levels are associated with the pathogenesis of autoimmune thyroid disorders during pregnancy in women [63]. Furthermore, pregnancy enhances the expression of the *C1r* gene in bovine endometrium, which is related to the regulation of the maternal immune system [64]. Moreover, IFN-γ can stimulate complement *C1rA* expression in mouse keratinocytes [64]. Serum complement C1r/C1s is increased in patients with preeclampsia, which is significantly correlated with the maternal uterine artery pulsatility index [65]. Compared with healthy controls, there is an increase in the serum concentrations of the C1r-C1s-C1 inhibitor complex in patients with untreated Graves’ disease [66]. IFN-γ enhances the expression of the *C1s* gene in mesenchymal stromal cells [67].

Protein and mRNA of thyroid C2 peaked at P16 in this study, and C3 protein and mRNA were upregulated at P16 and P25 compared to N16 and P13. The expression of C2 is greater on day 15 of pregnancy compared to day 15 of the estrous cycle in the porcine endometrium, and IFN-γ induces C2 expression in endometrial tissues [68]. In addition, complement *C2* mRNA and protein peak in the maternal lymph nodes at P16, which is due to the effect of IFNT and contributes to maternal immune regulation in ewes [62]. Additionally, blood complement C3 level has significant positive correlation with thyroid-stimulating hormone levels and body fat percentages in women with high body weight and body fat [69]. Moreover, complement *C3* mRNA and protein peak at P16 and then decrease at P25 in the maternal lymph nodes, which is related to the secretion of IFNT and pregnancy maintenance in sheep [62].

The data from this study showed that C4a and C5b expression in mRNA and protein levels peaked at P16, and C4a protein was strongly localized in the thyroid C-cells and capillaries. The plasma concentration of complement C4 protein is significantly higher in gravidas with preeclampsia compared to normal pregnancy during the first trimester [70]. In addition, human chorionic gonadotropin stimulates the expression of the *C4a* gene in the baboon endometrium, which is implicated in regulating the decidual immune environment during the window of implantation [71]. Furthermore, complement C5 is related to the development of atypical hemolytic uremic syndrome, and treatment with a C5 inhibitor can reduce the risk of pregnancy-associated atypical hemolytic uremic syndrome [72]. Moreover, the peak of C5b expression in the maternal thymus at P16 is implicated in preventing early embryonic lethality, and the decline of C5b expression at P25 is beneficial for pregnancy maintenance in sheep [61].

The data showed that early pregnancy induced peaks in mRNA and protein levels of C9 in the ovine thyroid at P16, and that C9 protein was strongly localized in the thyroid C-cells and capillaries. IFN-α treatment upregulates the serum concentration of C9 protein in patients with chronic hepatitis C [73]. In addition, the transcript level of the C9 gene in opossum uterine tissues peaked on embryonic days 13 and 14, and fell on post-natal day 1 and in nonpregnant past breeders [74]. Similar results have been reported, showing that *C9* mRNA and protein peak in maternal immune organs, including the thymus and lymph nodes, which is implicated in the maternal immunoregulation induced by IFNT [61,62].

In summary, the peaks of C1q, C1r, C2, C4a, C5b, and C9 at P16 may be attributed to the peak of IFNT, and changes in the expression of C1s and C3 may be related to the immune adaptation of the maternal thyroid to pregnancy and thyroid hormone secretion.

Despite the different pregnancy recognition molecular signals and implantation invasion depth between sheep and humans, the pre-implantation preparation of the uterine microenvironment and the initial dialogue at the embryo–maternal interface are remarkably conserved in both species. Thus, these results may provide references for human-related research. The molecular mechanism of early pregnancy modulating NF-κB and TLR signaling pathways and complement components in ovine maternal thyroid should be further confirmed by in vitro experiments using thyroid tissue explants, IFNT and/or progesterone. In addition, although the concentrations of IFNT and progesterone have been reported in many studies, they have not been measured in this study. Furthermore, it is necessary to further expand the sample size and perform research on thyroid hormone secretion during pregnancy. Despite these limitations, the results of this study support the findings of early pregnancy modulating these immune-related signaling pathways in the ovine maternal thyroid.

## 4. Materials and Methods

### 4.1. Animals and Experimental Design

Twenty-four multiparous ewes (small-tail Han sheep), aged 18 ± 0.5 months, with good reproductive function and similar body conditions (average weight of 41 ± 1.5 kg, body condition score of 3 ± 0.5), were selected for this study in October. The females were fed the same diet to meet the NRC (National Research Council, 2007) requirements under indoor feeding. The animals were randomly divided into four groups (*n* = 6 for each group) and were observed daily for estrus using vasectomized rams. After the detection of sexual receptivity (designated as day 0), the ewes from three groups were mated twice with intact rams at 12 h intervals, while the other group was not. Thyroid samples were taken on gestation days 13, 16, and 25 (P13, P16, and P25), as well as on day 16 of the estrous cycle (N16), immediately after the ewes were stunned followed by exsanguination (*n* = 6 for each group). Their pregnancy status was verified by observing the presence of the conceptus in the uterus. Following thyroid collection, transverse pieces of thyroid (0.5 cm^3^) were fixed in fresh 4% (*w*/*v*) paraformaldehyde in PBS buffer (pH 7.4), and the remaining portions were frozen in liquid nitrogen for subsequent mRNA and protein extraction/analysis.

### 4.2. RNA Extraction and qRT-PCR Assay

TRIzol (Invitrogen, Carlsbad, CA, USA) was used for RNA extraction according to the manufacturer’s instructions. NanoDrop^®^ (Thermo Fisher Scientific, Waltham, MA, USA) was used to measure the concentration of total RNA at 260/280 nm, and RNA integrity was verified through electrophoresis on 1% agarose gel. The cDNA was quantified using NanoDrop^®^ (Thermo Fisher Scientific). Reverse transcription was performed using the FastQuant RT Kit (Tiangen Biotech Co., Ltd., Beijing, China), and quantitative PCR was performed using the SuperReal PreMix Plus Kit (Tiangen Biotech) as described previously [47]. The primer sequences of the above immune signals, and *GAPDH* were listed in Table S1. The relative expression values of mRNA were assessed using the 2^−ΔΔCt^ analysis method, with *GAPDH* serving as an internal normalization control, and the relative expression value for the N16 group was set to 1.

### 4.3. Western Blot

The total proteins from thyroid samples were extracted using RIPA Lysis Buffer (Biosharp, Hefei, China; BL504A). Equal amounts of total proteins were electrophoresed through 12% polyacrylamide gels and electroblotted onto polyvinylidene fluoride membranes (Millipore, Bedford, MA, USA). Immunoreactive bands were probed with the primary antibodies listed in Table S2 at 4 °C overnight. The GAPDH antibody (sc-47724, Santa Cruz Biotechnology, Santa Cruz, CA, USA) was used to monitor sample loading. Secondary goat anti-mouse IgG-HRP (BL001A, Biosharp, Hefei, China) or goat anti-rabbit IgG-HRP (Biosharp, BL003A) was used at 1:2000 dilution for an hour at room temperature. An ECL western blotting detection reagent (Tiangen Biotech) was used to reveal protein bands with X-ray films. Quantity One V452 (Bio-Rad Laboratories, Hercules, CA, USA) was used to quantify the immunoreactive bands, and the relative abundances of the proteins were calculated using the loading control (GAPDH).

### 4.4. Immunohistochemistry Analysis

For immunohistochemistry analysis, endogenous peroxidase activity was eliminated by treating the samples with 3% H_2_O_2_, and 5% goat serum was added to the slices to block non-specific binding. Sections were then incubated with primary antibodies in a 1:100 dilution, including NF-κB1 and RelA antibodies for NF-κB subunits, IκBβ and IκBε antibodies for the IκB family, TLR2 and TRAF6 antibodies for the TLR signaling pathway, as well as C4a and C9 antibodies for complement components (Table S2), in a humidified chamber at 4 °C overnight. The secondary goat anti-mouse IgG-HRP (BL001A, Biosharp, Hefei, China) was incubated for an hour at room temperature at a 1:500 dilution. Sections were then observed using a DAB kit (Tiangen Biotech) and counterstained with hematoxylin. Finally, a light microscope (Nikon Eclipse E800, Tokyo, Japan) with a digital camera AxioCam ERc 5s was used to capture images, which were used to analyze the intensity of staining and density of stained cells by assigning an immunoreactive intensity on a scale of 0 to 3 (0: negative, 1: weak, 2: moderate, 3: strong) by 2 different investigators in a blinded fashion, as described previously [47].

### 4.5. Statistical Analyses

The data were subjected to a completely randomized design with subsampling for six animals per group using the Proc Mixed models of SAS (Version 9.1; SAS Institute, Cary, NC, USA). For thyroids from different stages, of gestation or pregnancy status, the model contained the random effects of ewes and fixed effects of stage of gestation, pregnancy status and the interaction of stage of gestation and pregnancy status. Data normality was tested using the PROC UNIVARIATE procedure in SAS version 9.2 (SAS Institute Inc.), and the data were found to be normally distributed. Comparisons among the relative expression levels of different groups were made using a Tukey test. Data are presented as mean ± S.E.M. Groups were considered significantly different at *p* < 0.05.

## 5. Conclusions

NF-κB and TLR signaling pathways and complement components are key immune regulatory signaling pathways. In this study, early pregnancy modulates the expression of NF-κB and TLR signaling pathways and complement components in the ovine thyroid. The peaks of expression in NF-κB subunits, IκB family, and TLR pathways, as well as C1q, C1r, C2, C4a, C5b, and C9, may be associated with the peak of IFNT secreted from the conceptus. The changes in the expression of C1s and C3 may be due to the combined action of IFNT and progesterone during early pregnancy. The higher levels of IκBβ, IκBε, TRAF6, IRAK1, and C3 at P25 may be associated with the maintenance of pregnancy. In conclusion, the expression of NF-κB and TLR signaling pathways and complement components in the ovine thyroid was modulated by IFNT and progesterone, contributing to the regulation of maternal thyroid immunity and thyroid hormone secretion in ewes. These results may provide references for human-related research. In addition, further research is needed to confirm the mechanisms of maternal immune tolerance and embryo implantation regulated through NF-κB and TLR signaling pathways and complement components.

## Data Availability

Data supporting the findings of this study are available within the paper.

## Supplementary Materials

The following supporting information can be downloaded at: https://www.mdpi.com/article/10.3390/ijms27114791/s1.

## Abbreviations

The following abbreviations are used in this manuscript:

AITD	Autoimmune thyroid disease
BCL-3	B-cell lymphoma-3
C1	Complement component 1
GAPDH	Glyceraldehyde-3-phosphate dehydrogenase
IFNT	Interferon-tau
IKK	IκB kinase
IRAK1	Interleukin 1 receptor associated kinase 1
IκB	Inhibitor of NF-κB
MyD88	Myeloid differentiation primary response gene 88
N16	Day 16 of the estrous cycle
NF-κB	Nuclear factor kappa B
P13	Day 13 of pregnancy
P16	Day 16 of pregnancy
P25	Days 25 of pregnancy
TLR	Toll-like receptor
TRAF6	Tumor necrosis factor receptor-associated factor 6
