# Supplementary material for: Modulation of NF-κB and TLR Signaling Pathways and Complement Components in Ovine Maternal Thyroid During Early Pregnancy
Source: Int J Mol Sci. 2026 May 26;27(11):4791. doi: 10.3390/ijms27114791 (PMC13256796; doi:10.3390/ijms27114791)
Supplement: Supplementary file 1 [file ijms-27-04791-s001.zip › Figure 1S Original Western Blot for Figures 1-4.pdf]

NF-κB signaling pathway

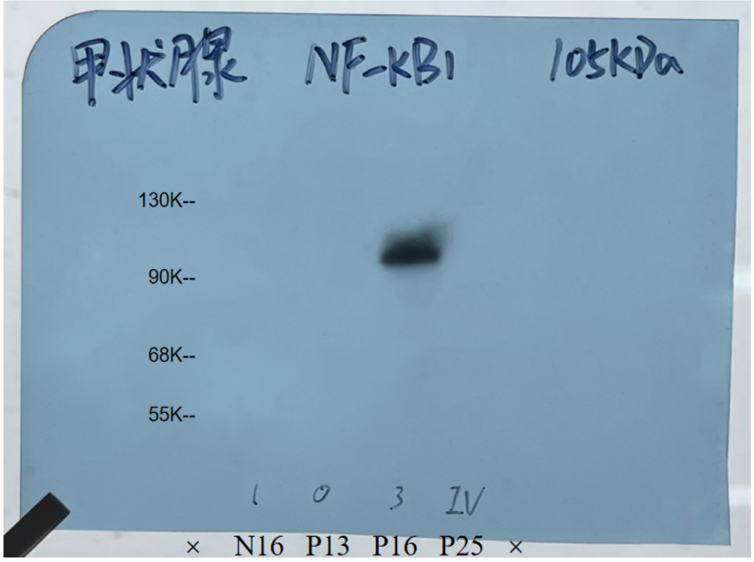

thyroid，NF-κB1

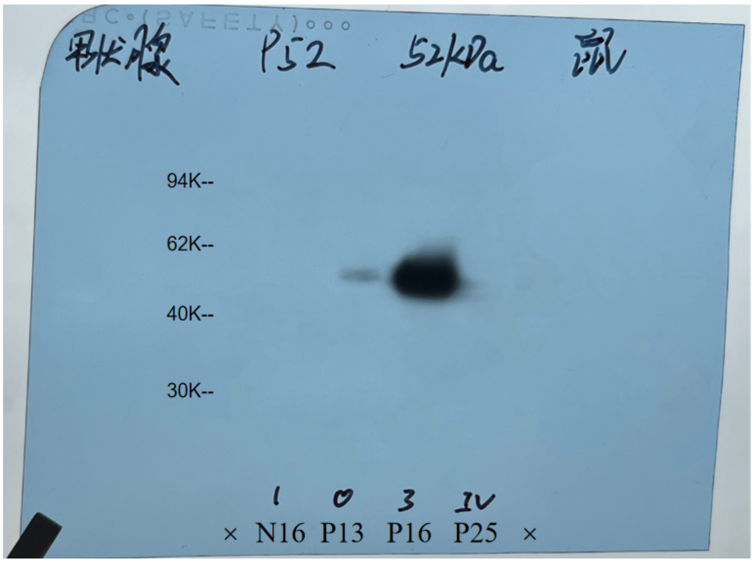

thyroid，NF-κB2(P52)

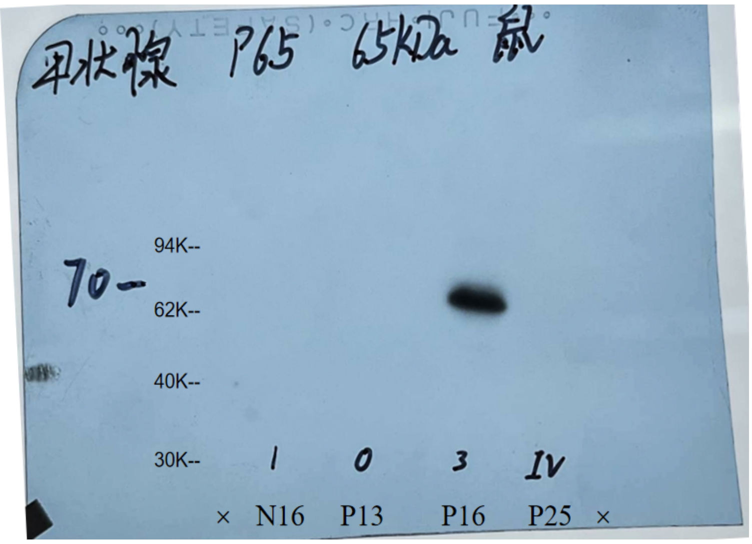

thyroid，RelA(P65)

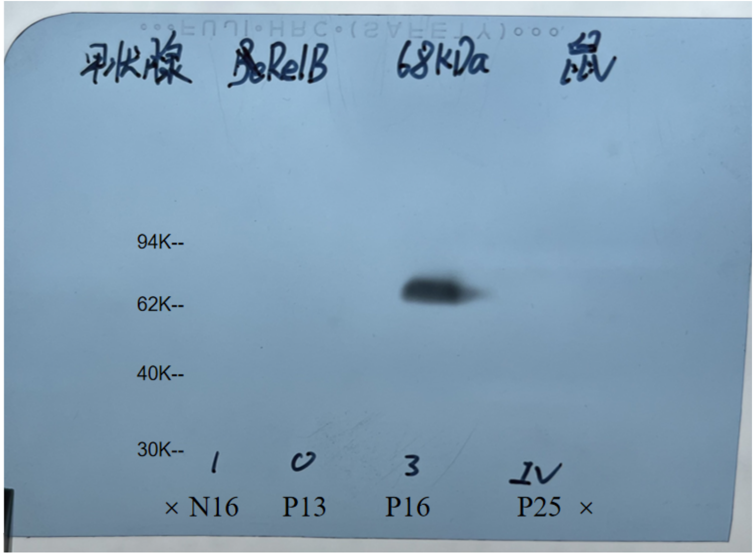

thyroid，RelB

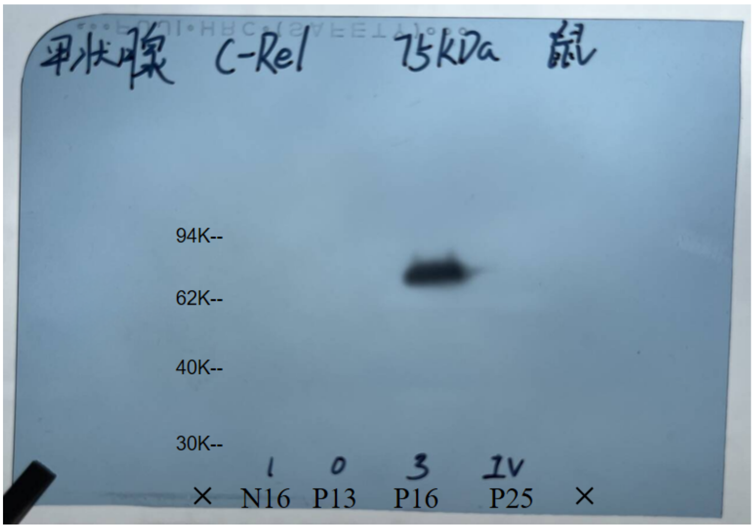

thyroid，c-Rel

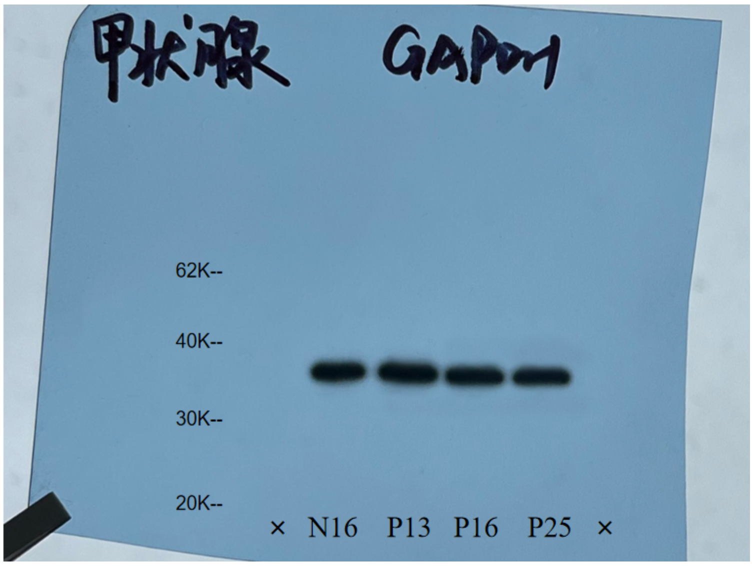

thyroid，NF-κB GAPDH

IκB family

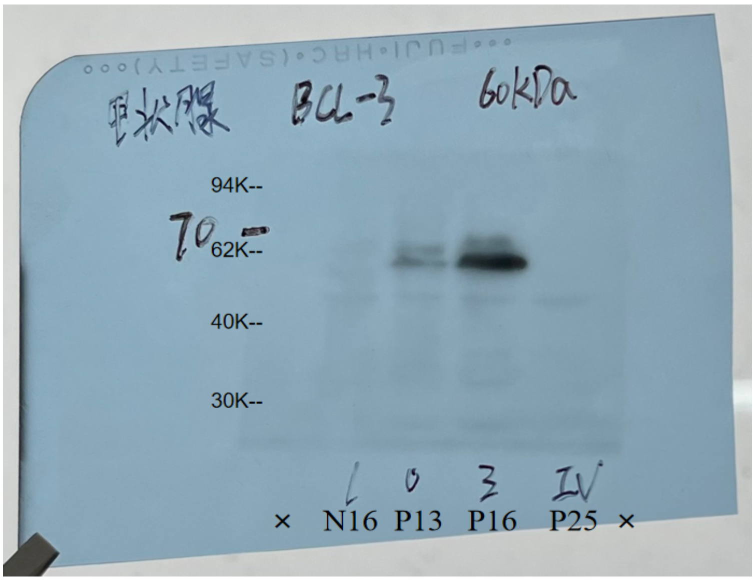

thyroid，BCL-3

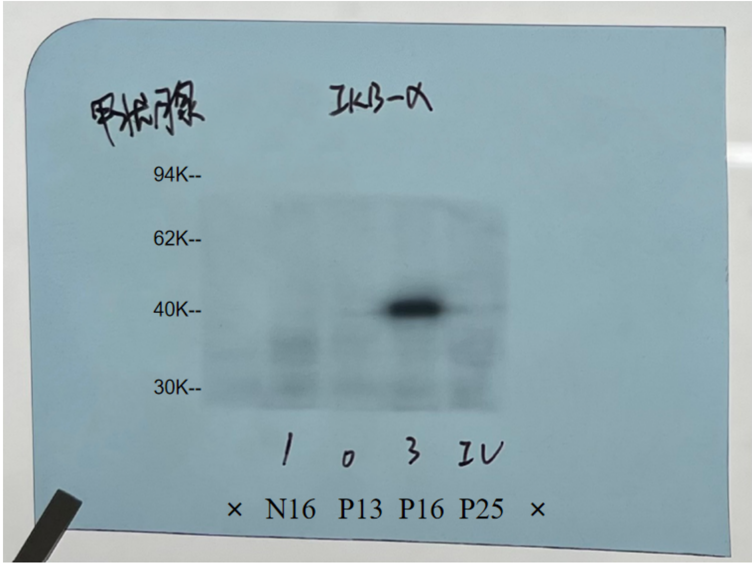

thyroid，IκBα

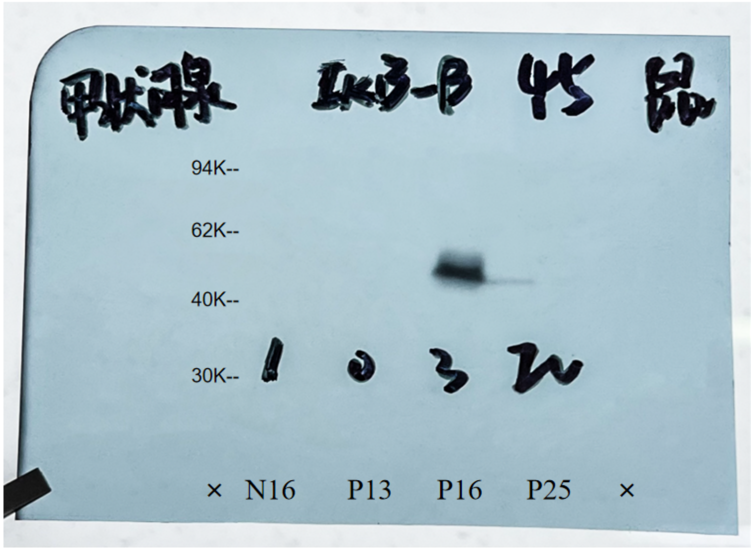

thyroid，IκBβ

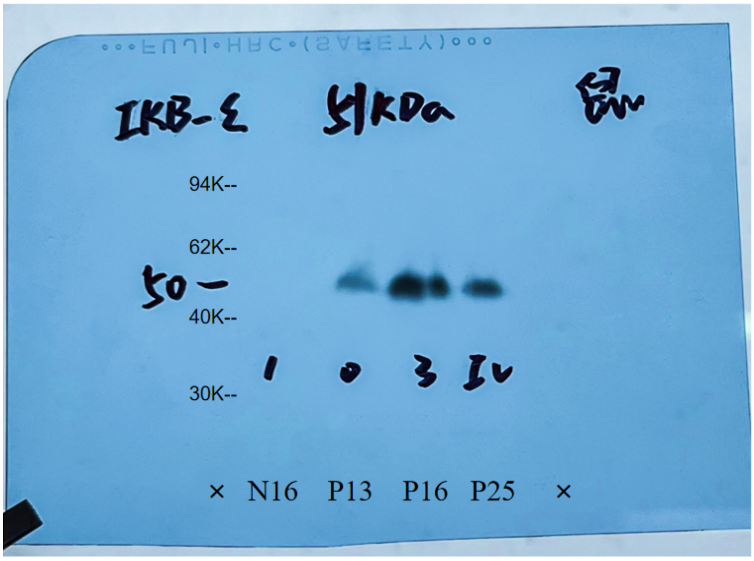

thyroid，IκBε

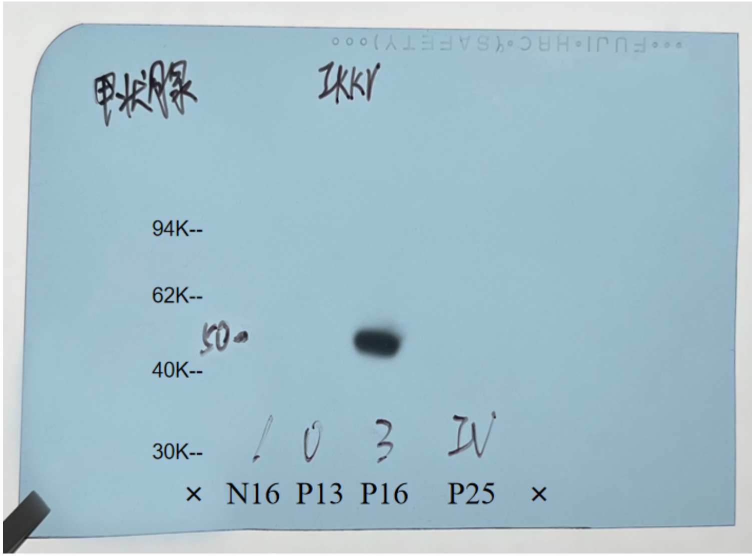

thyroid，IKKγ

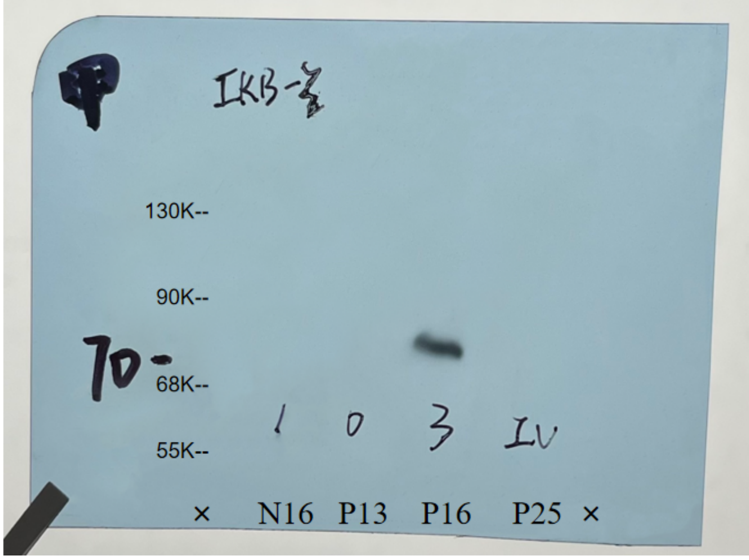

thyroid，IκBζ

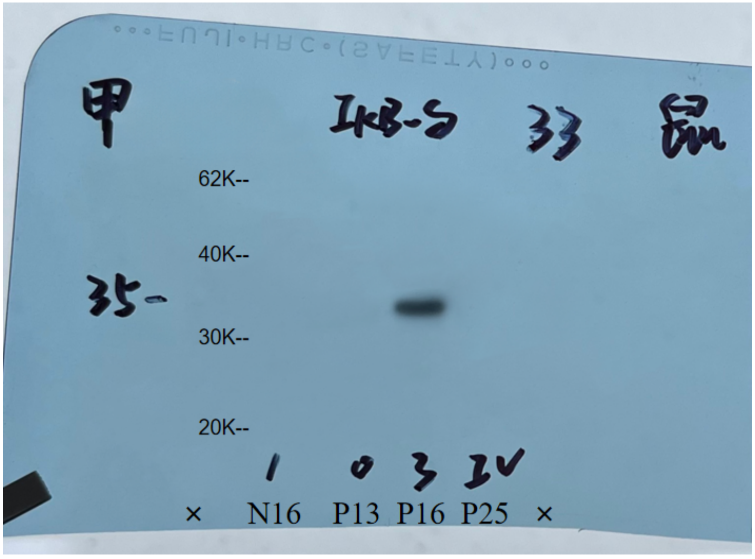

thyroid，IκBδ

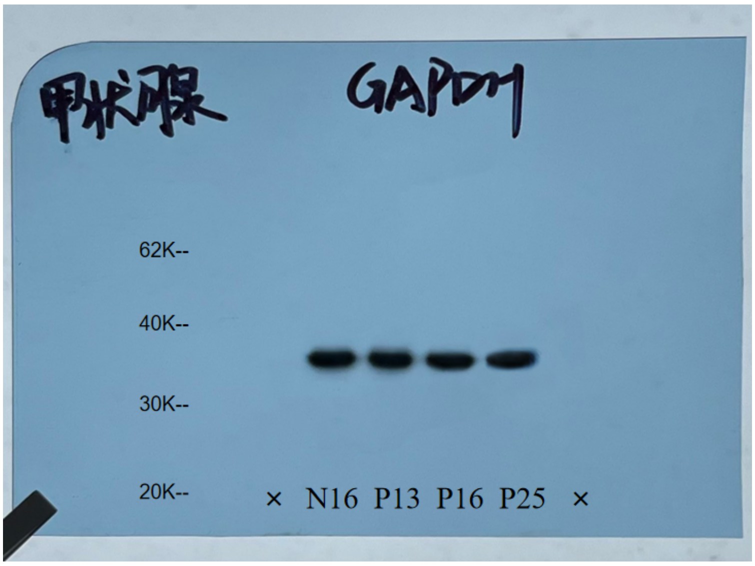

thyroid，IκB GAPDH

TLR signaling pathway

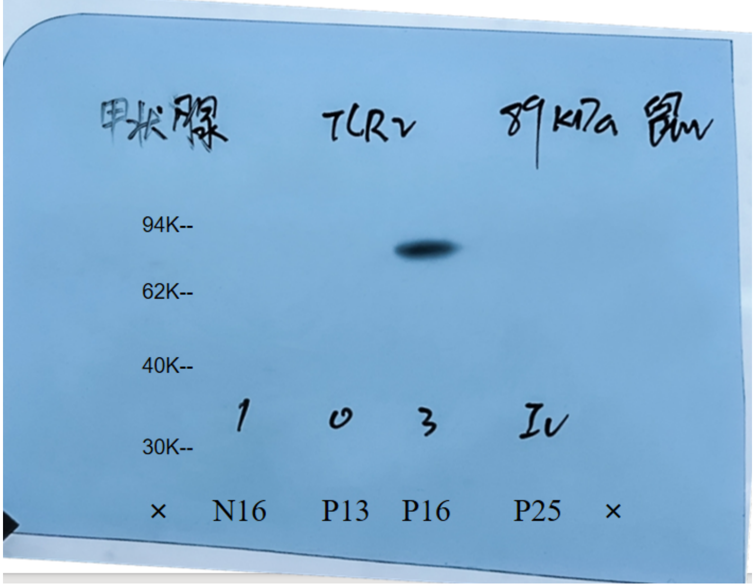

thyroid，TLR2

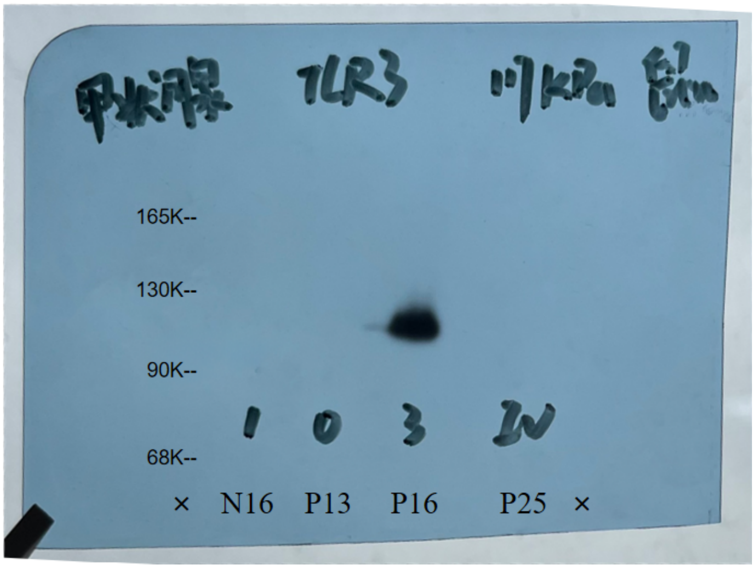

thyroid，TLR3

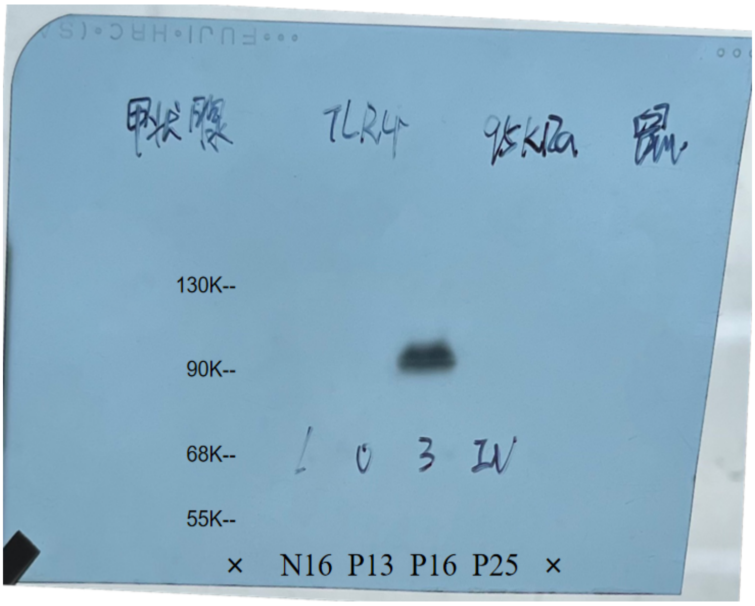

thyroid，TLR4

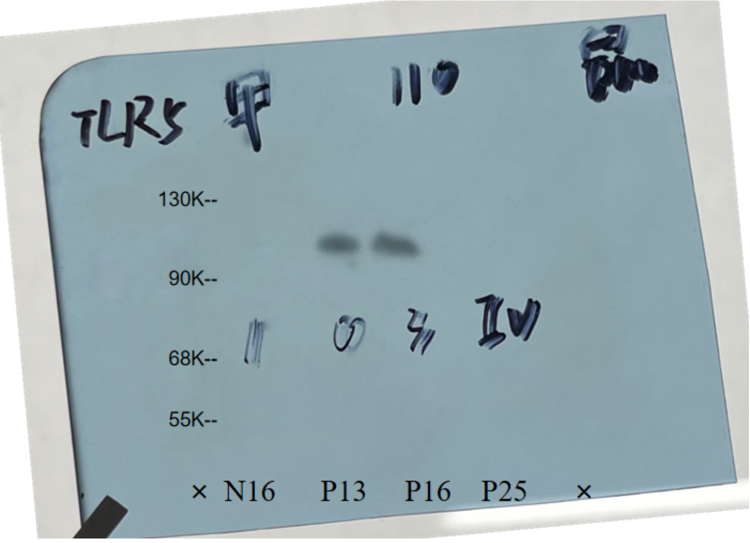

thyroid，TLR5

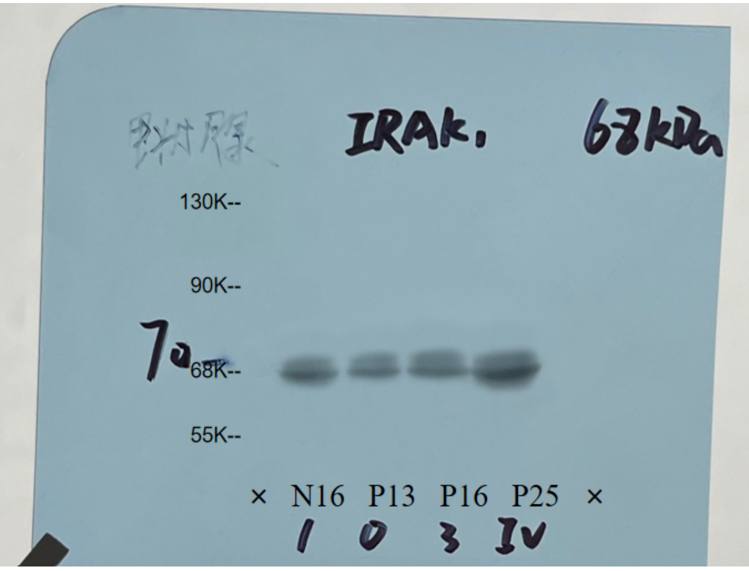

thyroid，IRAK1

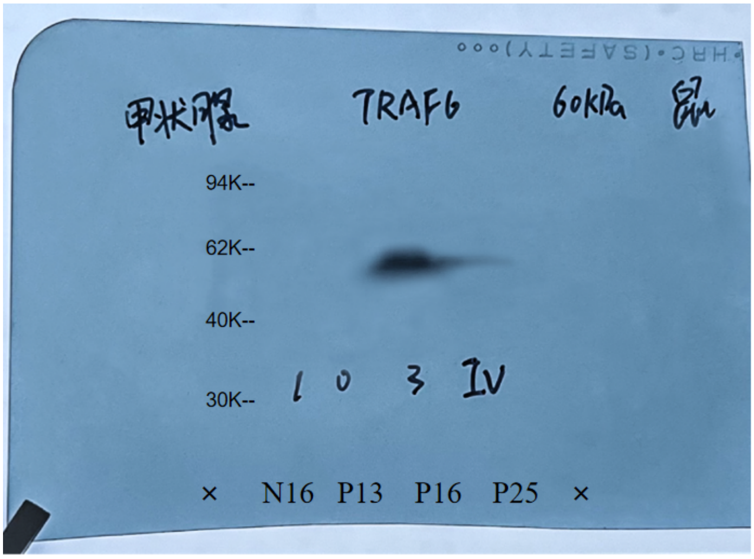

thyroid，TRAF6

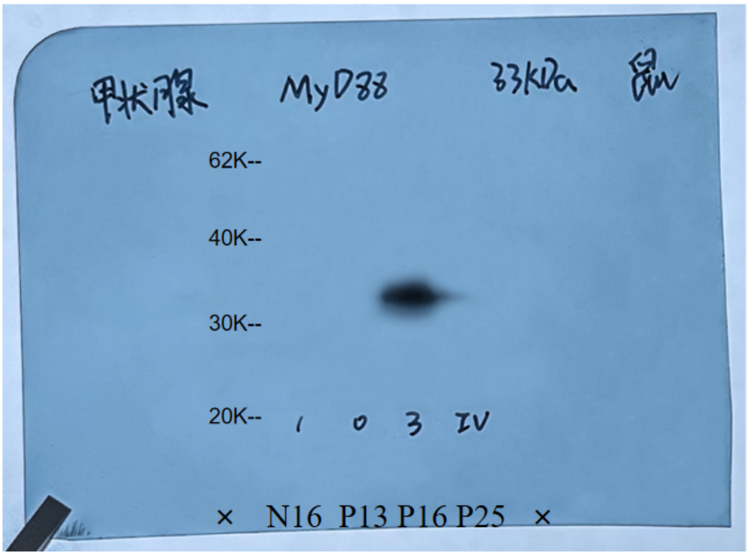

thyroid，MyD88

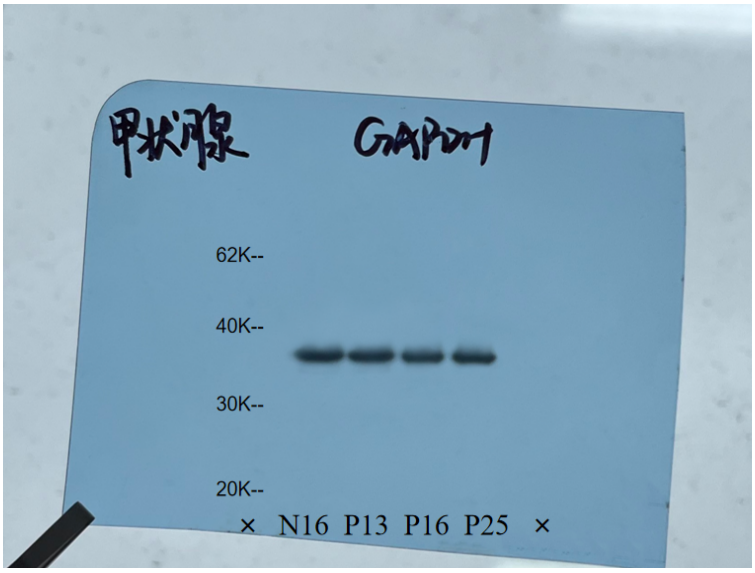

thyroid，TLR GAPDH

complement components

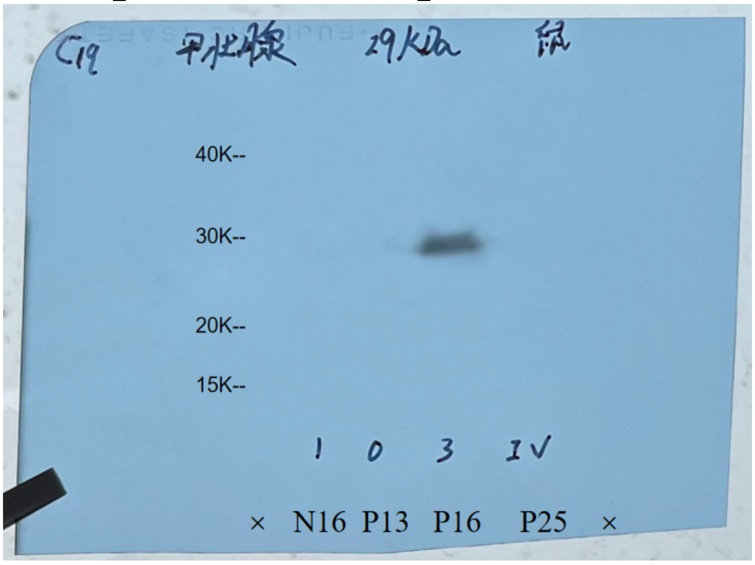

thyroid，C1q

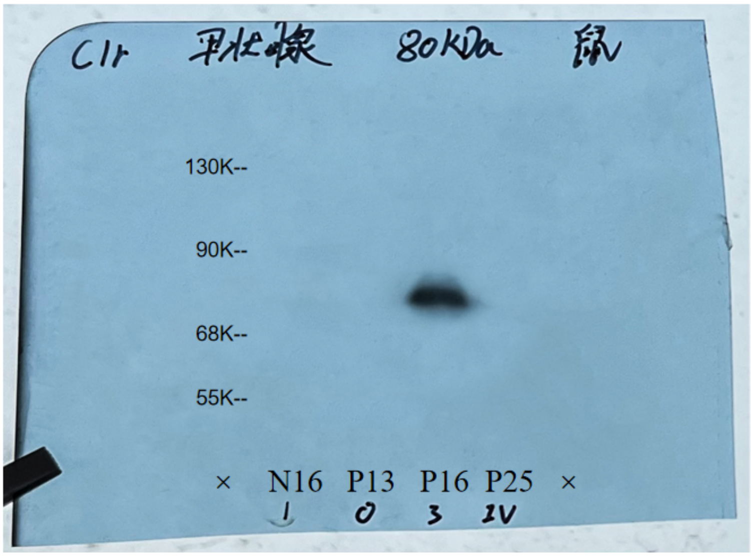

thyroid，C1r

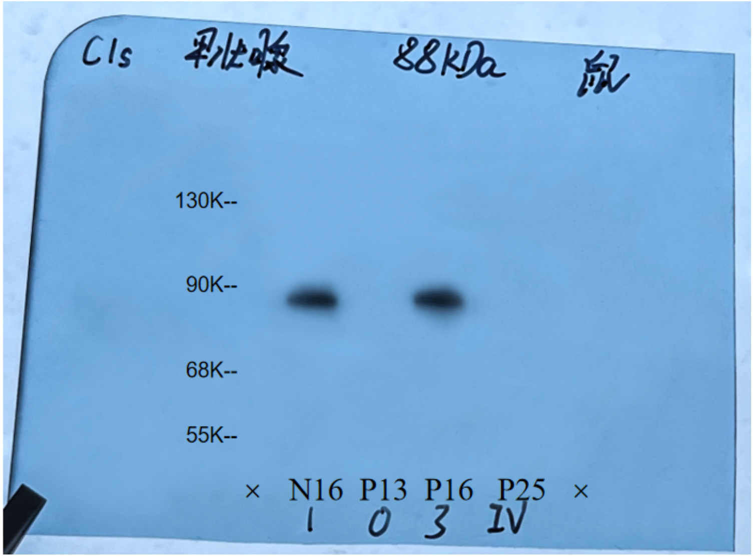

thyroid，C1s

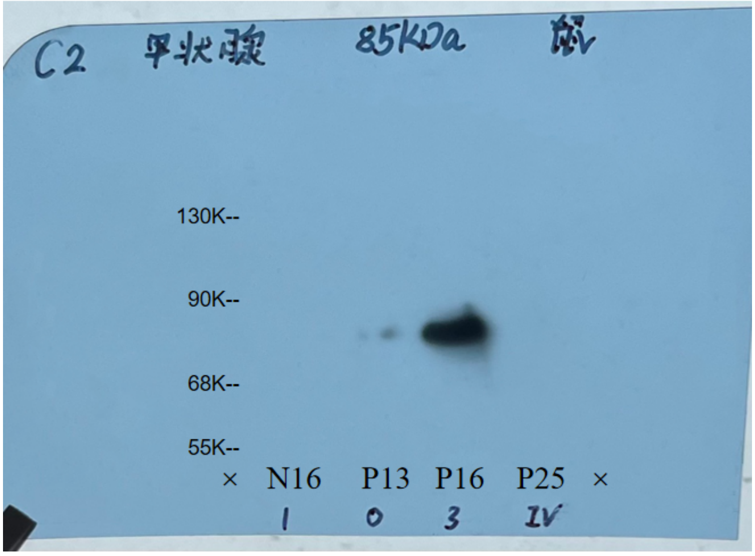

thyroid，C2

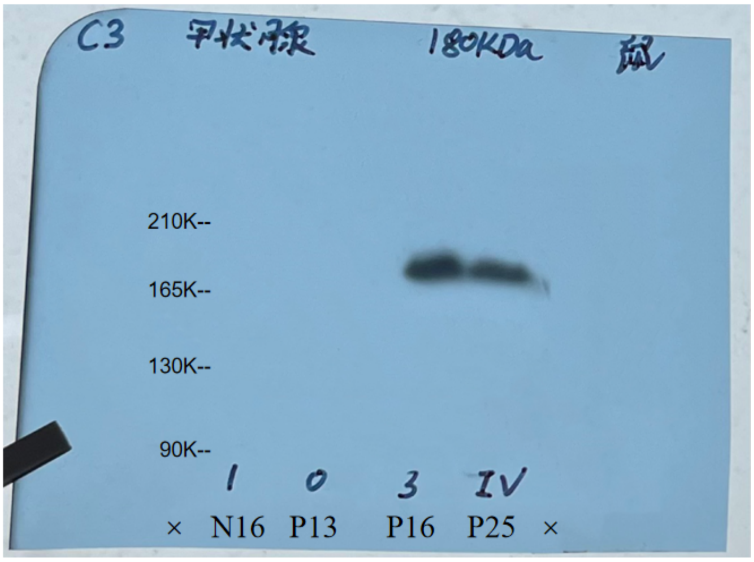

thyroid，C3

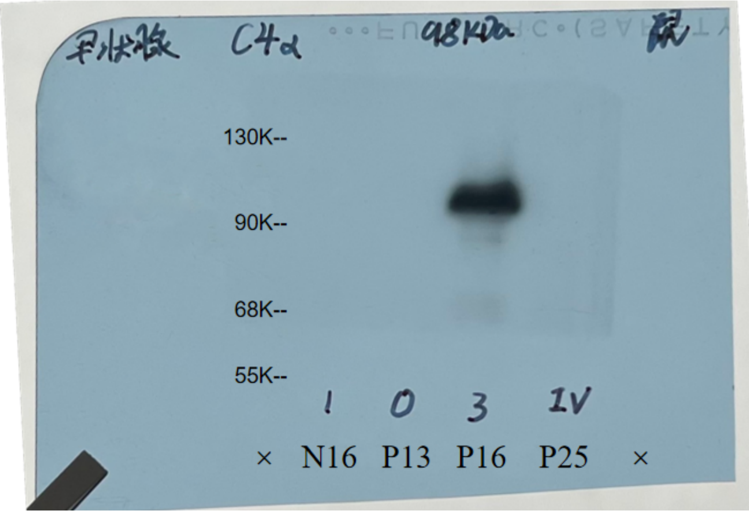

thyroid，C4a

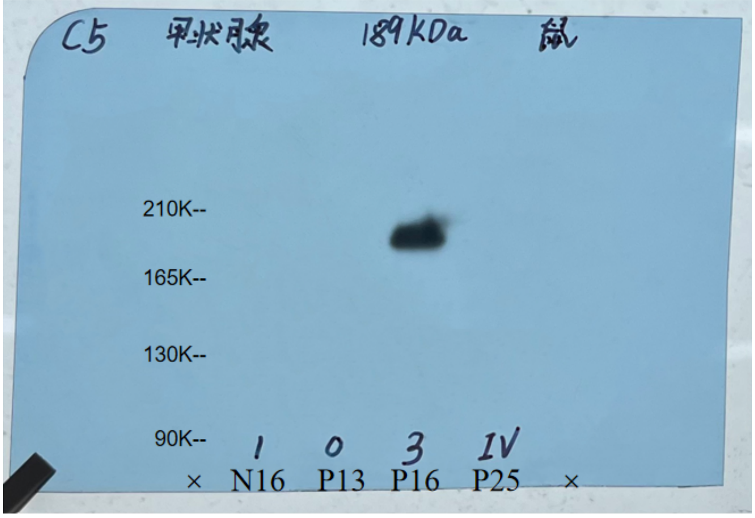

thyroid，C5b(C5)

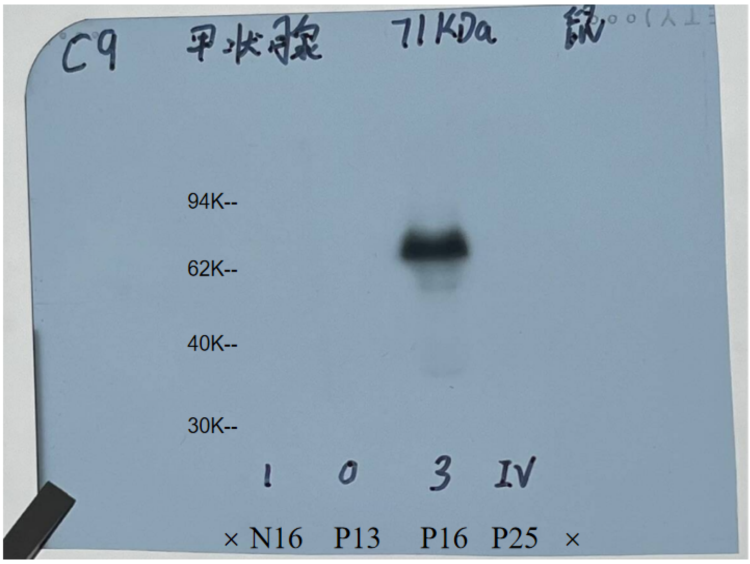

thyroid，C9

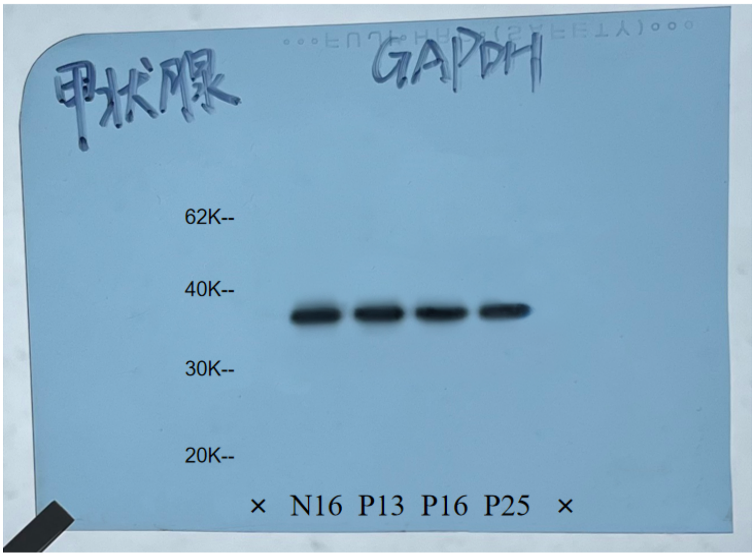

thyroid，complement GAPDH
