# Supplementary material for: Modulation of NF-κB and TLR Signaling Pathways and Complement Components in Ovine Maternal Thyroid During Early Pregnancy
Source: Int J Mol Sci. 2026 May 26;27(11):4791. doi: 10.3390/ijms27114791 (PMC13256796; doi:10.3390/ijms27114791)
Supplement: Supplementary file 1 [file ijms-27-04791-s001.zip › Supplementary.pdf]

Table S1 Primers used for RT-qPCR

Gene	Primer	Sequence	Size (bp)	Accession numbers
NF-κB1	Forward	CAAGCACAAGAAGGCAGCACAAC	113	XM_027970852.2
	Reverse	CAGCCATCAGCAGCAGCAGAC		
NF-κB2	Forward	GCCTGCTGAATGCCCTGTCTG	146	XM_042238744.1
	Reverse	CTCTGTTTCCTGTTCCACCGACTG		
RelA	Forward	TGGCGAGAGGAGCACAGACAC	92	XM_027959295.2
	Reverse	TGACCAGGGAGATGCGGACTG		
RelB	Forward	CGCTGACCTCTCCTCGCTCTC	93	XM_015100238.3
	Reverse	AAGCCGAAGCCATTCTCCTTGATG		
c-Rel	Forward	TCCTCCTCTGCGTCCATCTCAAG	104	XM_004005929.4
	Reverse	GTGGGGTGGGCGATTGATGAC		
BCL-3	Forward	GCGACCAGAGGCAATTTACTACCAG	98	XM_027978453.2
	Reverse	GAGGTGTAGGCAAGTTCAGCAGAG		
NFKBIA	Forward	AGGACGAGGAGTATGAGCAGATGG	130	NM_001166184.1
	Reverse	GCCAAGTGCAGGAACGAGTCTC		
NFKBIB	Forward	CCCCAAGACCTACCTCGCTCAG	119	XM_027978262.2
	Reverse	TCCAGTCCTCTTCACTCTCATCCTC		
NFKBIE	Forward	GCACTCACGTACATTTCCGAGGAC	97	XM_042236979.1
	Reverse	GCAGCAGAGCCAGGCAATACAG		
IKBKG	Forward	GGGCAACCAGAGGGAGGAGAAG	146	XM_027963334.2
	Reverse	GGCATGTCTTCAGGCGTTCCAC		
NFKBIZ	Forward	GCAAAGGCGTACAATGGAAACACC	137	NM_001306117.1
	Reverse	GGCTGCTCGTTCTCCAAGTTCC		
NFKBID	Forward	ACATTCGTGAGCATAAGGGCAAGAC	114	XM_027977435.2
	Reverse	GATGGTCAGTGGCATTGGGTTCC		
TLR2	Forward	GGAGCGAGTGGTGCAAGTATGAG	200	NM_001048231.1
	Reverse	CCAGAATGCTTCCTGCTGAGTCTC		
TLR3	Forward	TCTCTGAACAATGCCAAGCTGAGC	123	NM_001135928.1
	Reverse	GGTCGTGTGGCTGATTGTGTCC		
TLR4	Forward	TGTGAAGGACATGCCAGTGCTTG	80	NM_001135930.1
	Reverse	TGACAACCGACACGCTGATGATC		
TLR5	Forward	CGTGTGGAGCAGCAGGAAGAC	116	NM_001135926.1
	Reverse	CGCCGTTGAGGTCAGCTAAGC		
MyD88	Forward	GCGAGGACGTGCTGATGGAAC	82	NM_001166183.1

	Reverse	GATGCCTCCTGCTGCTGCTTC		
TRAF6	Forward	AACTGAGGCATCTTGAGGAGCATC	82	XM_015101111.2
	Reverse	TTCTGGAAGAGACGCTGGCATTG		
IRAK1	Forward	GCTACTGTGCTCAGAGTGGCTTC	82	XM_027962961.1
	Reverse	TGGACGTGGAGGCGGTCTTC		
C1qA	Forward	CAGGAGAACGTGTACCAGAGCAAC	122	XM_012152629.2
	Reverse	CTCCGAGAGGACCTGATGGACAG		
C1r	Forward	CCCAGACTACCGCCAGGAAGAG	109	XM_012175492.2
	Reverse	TGGGAGGCAGATTGGCAGGAG		
C1s	Forward	CCTGGCAAGTCTTCTTCTCGAACC	130	XM_004006917.4
	Reverse	ACCACTGAGGAGGACCCAACATAC		
C2	Forward	CCACCAATCCCATCCAGCAGAAG	95	XM_027958809.1
	Reverse	GGCGTCCAGGAGCAGGTAGAG		
C3	Forward	CGCCACCAGCAGACTATAACGATC	105	XM_027969774.1
	Reverse	AGCAGCCTTGACCTCCACCTC		
C4α	Forward	TTCAGGACAGGTGGTGAGAGGATC	167	XM_027958803.1
	Reverse	GGAGGAGATGGAGGCGACAGAG		
C5	Forward	GCTACGCTGGTGTTACTCTGGATC	157	XM_004003966.3
	Reverse	GCAGACATGACCTCGCCTATAAGC		
C9	Forward	GCCGCAACAGAGTGGTGGAAG	138	XM_004017026.3
	Reverse	TGCCATCCCTAACTCGGTCACAG		
GAPDH	Forward	GGGTCATCATCTCTGCACCT	176	NM_001190390.1
	Reverse	GGTCATAAGTCCCTCCACGA		

Table S2 Antibodies used for western blot and immunohistochemistry

Description	Number	Source
Mouse anti-NF-κB1 monoclonal antibody	sc-8414	Santa Cruz Biotechnology, Santa Cruz, CA, USA
Mouse anti-NF-κB2 monoclonal antibody	sc-7386	Santa Cruz Biotechnology, Santa Cruz, CA, USA
Mouse anti-RelA monoclonal antibody	sc-8008	Santa Cruz Biotechnology, Santa Cruz, CA, USA
Mouse anti-RelB monoclonal antibody	sc-166416	Santa Cruz Biotechnology, Santa Cruz, CA, USA
Mouse anti-c-Rel monoclonal	sc-6955	Santa Cruz

antibody		Biotechnology, Santa Cruz, CA, USA
Mouse anti-BCL-3 monoclonal antibody	sc-32741	Santa Cruz Biotechnology, Santa Cruz, CA, USA
Mouse anti-IκBα monoclonal antibody	sc-1643	Santa Cruz Biotechnology, Santa Cruz, CA, USA
Mouse anti-IκBβ monoclonal antibody	sc-390622	Santa Cruz Biotechnology, Santa Cruz, CA, USA
Mouse anti-IκBε monoclonal antibody	sc-7275	Santa Cruz Biotechnology, Santa Cruz, CA, USA
Mouse anti-IKKγ monoclonal antibody	sc-166398	Santa Cruz Biotechnology, Santa Cruz, CA, USA
Rabbit anti-IκBζ polyclonal antibody	ab155142	Abcam, Cambridge, UK
Rabbit anti-NFKBID polyclonal antibody	ab232913	Abcam, Cambridge, UK
Rabbit anti-TLR2 polyclonal antibody	ab191458	Abcam, Cambridge, UK,
Mouse anti-TLR3 monoclonal antibody	sc-32232	Santa Cruz Biotechnology, Santa Cruz, CA, USA
Mouse anti-TLR4 monoclonal antibody	sc-293072	Santa Cruz Biotechnology, Santa Cruz, CA, USA
Mouse anti-TLR5 monoclonal antibody	sc-517439	Santa Cruz Biotechnology, Santa Cruz, CA, USA
Mouse anti-MyD88 monoclonal antibody	sc-136970	Santa Cruz Biotechnology, Santa Cruz, CA, USA
Mouse anti-TRAF6 monoclonal antibody	sc-8409	Santa Cruz Biotechnology, Santa Cruz, CA, USA
Rabbit anti-IRAK1 polyclonal antibody	ab137327	Abcam, Cambridge, UK
Mouse anti-C1q monoclonal antibody	sc-53544	Santa Cruz Biotechnology, Santa Cruz, CA, USA
Mouse anti-C1r monoclonal antibody	sc-514105	Santa Cruz Biotechnology, Santa Cruz, CA, USA
Mouse anti-C1s monoclonal antibody	sc-365273	Santa Cruz Biotechnology, Santa Cruz, CA, USA
Mouse anti-C2 monoclonal antibody	sc-373809	Santa Cruz Biotechnology, Santa Cruz, CA, USA
Mouse anti-C3 monoclonal antibody	sc-28294	Santa Cruz Biotechnology, Santa

			Cruz, CA, USA
Mouse anti-C4a antibody	monoclonal	sc-271181	Santa Cruz Biotechnology, Santa Cruz, CA, USA
Mouse anti-C5b antibody	monoclonal	sc-398247	Santa Cruz Biotechnology, Santa Cruz, CA, USA
Mouse anti-C9 antibody	monoclonal	sc-390000	Santa Cruz Biotechnology, Santa Cruz, CA, USA
Mouse anti-GAPDH antibody	monoclonal	sc-47724	Santa Cruz Biotechnology, Santa Cruz, CA, USA
